# Designing Formulae for Ranking Search Results: Mixed Methods Evaluation Study

**DOI:** 10.2196/30258

**Published:** 2022-03-25

**Authors:** Laura Douze, Sylvia Pelayo, Nassir Messaadi, Julien Grosjean, Gaétan Kerdelhué, Romaric Marcilly

**Affiliations:** 1 Inserm, Centre d'Investigation Clinique pour les Innovations Technologiques 1403 Institut Coeur-Poumon Lille France; 2 Unité Labellisée de Recherche 2694 - METRICS : Évaluation des technologies de santé et des pratiques médicales Univ. Lille, Centre Hospitalier Universitaire de Lille Lille France; 3 Département de médecine générale Univ. Lille Lille France; 4 Département d’Informatique et d’Information Médicales Centre hospitalier universitaire de Rouen Rouen France; 5 Laboratoire d'Informatique Médicale et d'Ingénierie des Connaissances en e-Santé, Inserm 1142 Sorbonne Paris Nord University Sorbonne Paris Cité Villetaneuse France

**Keywords:** information retrieval, search engine, topical relevance, search result ranking, user testing, human factors

## Abstract

**Background:**

A major factor in the success of any search engine is the relevance of the search results; a tool should sort the search results to present the most relevant documents first. Assessing the performance of the ranking formula is an important part of search engine evaluation. However, the methods currently used to evaluate ranking formulae mainly collect quantitative data and do not gather qualitative data, which help to understand what needs to be improved to tailor the formulae to their end users.

**Objective:**

This study aims to evaluate 2 different parameter settings of the ranking formula of LiSSa (the French acronym for *scientific literature in health care*; Department of Medical Informatics and Information), a tool that provides access to health scientific literature in French, to adapt the formula to the needs of the end users.

**Methods:**

To collect quantitative and qualitative data, user tests were carried out with representative end users of LiSSa: 10 general practitioners and 10 registrars. Participants first assessed the relevance of the search results and then rated the ranking criteria used in the 2 formulae. Verbalizations were analyzed to characterize each criterion.

**Results:**

A formula that prioritized articles representing a consensus in the field was preferred. When users assess an article’s relevance, they judge its topic, methods, and value in clinical practice.

**Conclusions:**

Following the evaluation, several improvements were implemented to give more weight to articles that match the search topic and to downgrade articles that have less informative or scientific value for the reader. Applying a qualitative methodology generates valuable user inputs to improve the ranking formula and move toward a highly usable search engine.

## Introduction

### Background

The evolution of the World Wide Web from a static network (Web 1.0) to a semantic web (Web 3.0) is ever more palpable [1]. The semantic web provides access to information in billions of heterogeneous documents in various formats, stored on different operating systems, and references among others to varying extents. This opens up a range of possibilities such as facilitating rapid access to targeted data [1]. However, the challenge for health care professionals is to identify relevant documents in this ocean of data [2,3].

In this context, search engine evaluation and improvement are key issues [4]. As soon as the discipline of information retrieval was established, researchers started to combine structured evaluation methods. For example, the Cranfield method (developed in 1962 [5]) soon became a benchmark for evaluating information retrieval and the Text Retrieval Conference has encouraged initiatives in information retrieval since 1992 [6].

Since then, the methods for evaluating information retrieval have diversified to meet a broader range of objectives. There are two main types of evaluation: system-oriented evaluations [4,7] that focus on search engine optimization (search efficiency, recall, accuracy, etc) and user-oriented evaluations [4,7,8] that seek to improve the user experience and search engine’s value (usability, expressivity, relevance, etc). One of the most important factors, perhaps the most important factor for search engines, is the relevance of search results [9].

There are two main definitions of relevance [10]: objective relevance (ie, the search result contains the submitted keyword) and subjective relevance (ie, the search result satisfies the user). Subjective relevance can then be subdivided into four main categories [10]: topical, situational, motivational, and affective. Topical relevance is the most studied type [4,8] and is the subject of this study; it was defined by Harter [11] as “how well the topic of the information retrieved matches the topic of the request.”

It is possible to evaluate topical relevance by involving users (eg, when relevance is rated by one or more expert or nonexpert participants) [12,13] or without their involvement (eg, in batch evaluations, such as the Cranfield method). Conventional methods for evaluating the relevance or performance of search engines are mostly based on comparisons between several formulae or a comparison with a gold standard. These comparisons are performed with quantitative data (mostly judges’ ratings) [4,14,15]. This method generates a large amount of data. The evaluation is quick and can be performed remotely. Thus, it is possible to include a large number of judges and test a large number of search queries. However, this method does not provide qualitative data, information on why a formula fails, or information on how to improve a formula’s performance.

Some studies have included user feedback, that is, the collection of qualitative data on perceived relevance and judgment criteria [16,17]. However, to the best of our knowledge, most of these studies sought to model and understand users’ relevance judgments rather than to evaluate and improve existing ranking formulae. This is a shortcoming of current methods for improving sorting formulae. Qualitative methods should also be used to identify the strengths and weaknesses of formulae.

In human factors research, it is well known that participative methods (notably user-centered designs involving users at each step in the design process [18]) improve the usability of a product before implementation in real settings. If users are not involved in the design process, their needs are often hypothetical and come from designers’ own representations of the field [19]. The tools thus created may not correspond to the users’ true needs and habits, which typically creates usability problems. Iterative evaluations are needed to improve effectiveness, efficiency, utility, acceptability, end user satisfaction, and (in health care) the safety of health care professionals and patients [20-22]. A proven method is user testing (also known as usability testing), which “calls for representative users to perform representative tasks as a means to reveal the interactive strengths and opportunities for improvement of a device” [23]. When coupled with the think-aloud method, a verbal report method from cognitive psychology that provides information on the cognitive behavior of participants performing a task [24], user testing collects valuable qualitative data about users’ behaviors and needs. Given that the user and moderator can interact during the evaluation, the user’s behavior and verbalizations can be investigated directly and may help clarify the user’s responses.

With a view to prompting further design innovations, we describe here the formative assessment of the *sort by relevance* function of a health care literature search engine. Taking a broader view, we developed a 2-step methodology that lies between a conventional information retrieval approach (for evaluating the relevance of search results) and a conventional human factors approach (for evaluating the usability of a new technology). With the objective of improving the ranking and moving toward a useful, usable search interface, we collected data on the performance of 2 *sort by relevance* formulae and on their strengths and weaknesses. We focused on the value of active end user involvement in this evaluation as a means of improving topical relevance in comparison with common evaluation methods used in the field.

### Study Context

This study was part of a broader research program funded by the French National Research Agency. The objective of the project is to develop a health care literature search engine *LiSSa*, the French acronym for *scientific literature in health care* (LiSSa.fr [25]). The particularity of the search engine is that both the interface and content are entirely in French.

PubMed is the most widely used search engine for scientific literature on health care. It is an important tool for all health professionals, for lifelong training and for updating their knowledge. However, English is a hindrance to reading by many French health care professionals [26]. French professionals are often not sufficiently fluent in English to read scientific articles. For these professionals, the lack of tools in French that allows them to find scientific literature in their native language is an obstacle to updating their medical knowledge and continuing education [26]. LiSSa is a French language tool that provides access to French scientific literature on health to people who are not specialized in scientific research and who do not understand English well enough. The main target users are general practitioners (GPs) and hospital registrars for continuing education, updating knowledge, and helping them find scientific articles to solve medical issues. In short, the tool helps them in the context of daily practice outside any academic or institutional environment. LiSSa currently encompasses over 1,300,000 French scientific references provided by various publishers and sources, among which the PubMed database (US National Library of Medicine [NLM]) accounts for 53% and publisher Elsevier accounts for 23% (18% without overlap).

**Figure 1:**
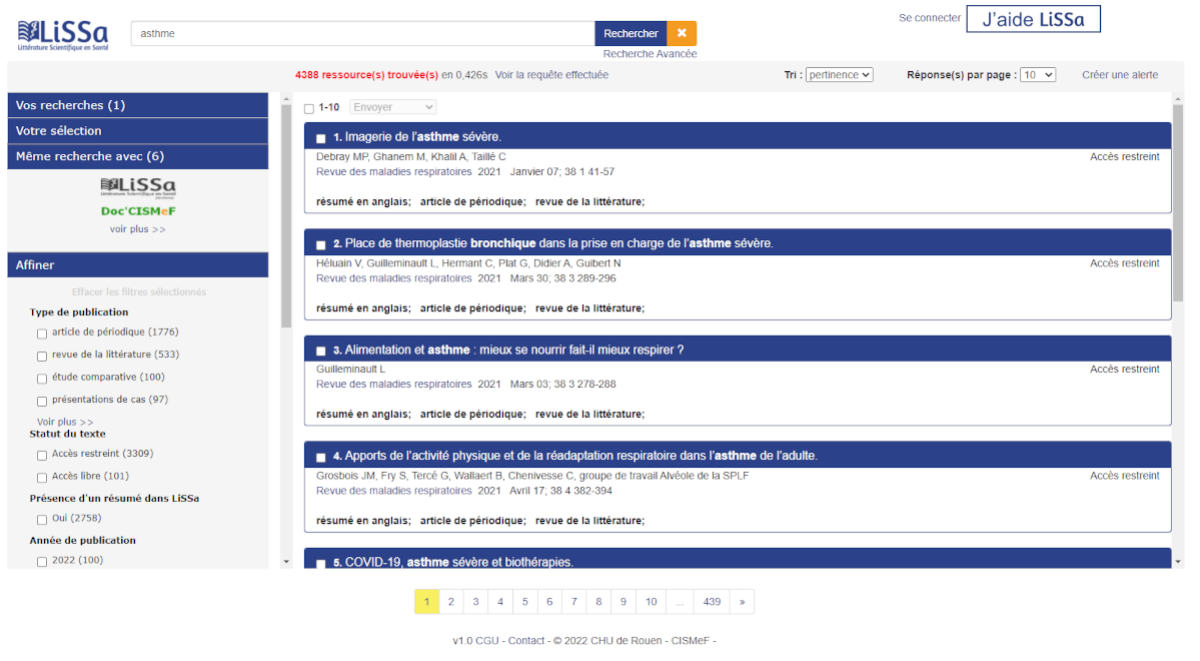
A screenshot of a search results page of LiSSa.fr [25].

The project is led by the Department of Medical Informatics and Information (D2IM). This department from the Rouen University hospital specializes in eHealth, more precisely in knowledge representation (terminologies, ontologies, etc) and information management (databases and search engines). The D2IM design team comprises physicians, librarians, and computer scientists. Their previous work includes Catalogue and Index of French Language Health Resources on the Internet and the Health Terminology and Ontology Portal [27,28], both available on the web. D2IM created the LiSSa database and designed the graphical user interface for the tool. The other academic partner is the Clinical Investigation Centre for Innovative Technology (for *Clinical Investigation Centre for Innovative Technology* in French) of the Lille University Hospital, an academic research laboratory that works to improve the design and evaluation of innovations in health care and is responsible for the usability assessments of the tool. In total, 3 companies were partners in the project: Elsevier Masson, one of the world’s leading science publishers, and the French start-ups Alicante and Sensegate. The LiSSa.fr [25] website (Figure 1) was published in 2014 [29]. Initial evaluations by GPs revealed a lack of relevance to the search results. They considered that the sorted results did not present the most relevant articles first; the top-ranked articles were often of little practical value, too old, or not representative of the topic. Specific work to improve the sorting of results needs to be conducted.

The most commonly used search engines use several criteria to rank database search results: the match between the keywords used and the metadata, the number of views, and contextual data (such as the user’s previous search or geographic location) [30-32]. However, the best results are typically obtained using a combination of ranking criteria. With regard to the LiSSa search engine, D2IM considered two ranking formulae, A and B, which differed in the weight attributed to the same set of criteria:

- Formula A prioritizes recent novel articles by assigning more weight to the publication year.
- Formula B prioritizes general articles that represent consensus in the field (literature reviews, meta-analyses, etc) by assigning more weight to the publication type.

The formulae’s weighting criteria are listed in Table 1. All these criteria are based on metadata retrieved from publishers and PubMed (produced by the NLM) [26]. For papers not indexed in PubMed, metadata were automatically generated with a set of indexing terms from the NLM’s controlled vocabulary, used to index articles in the biomedical field (MeSH [Medical Subject Headings] thesaurus) [33].

**Table 1:** Weighting of each criterion in the ranking formulae A and B.

Criterion	Weighting for formula A	Weighting for formula B
Title	10	10
Subtitle	10	10
Author keywords	5	5
Major MeSH^a^ terms^b^	4	4
Minor MeSH terms	1	1
Nonexploded indexing	3	3
Exploded indexing^c^	1	1
Manual indexing^d^	3	3
Automatic indexing	1	1
Year of publication	10 for the current year and −2 for each year in the past	10 for the current year and −0.6 for each year in the past
Type of publication; for example, good practice guidelines, consensus statements, directives, literature reviews, and meta-analyses	0	3

^a^MeSH: Medical Subject Headings.

^b^In the field of biomedicine, articles are often indexed according to the MeSH thesaurus. LiSSa considers the MeSH terms to be major when they correspond to one of the article’s main themes or minor when they correspond to one of the article’s subthemes.

^c^The MeSH thesaurus is structured like a tree; an MeSH term typically has several hierarchical levels above and below it. For example, *asthma* belongs to the *bronchial diseases* category and one of its narrower terms is *status asthmaticus*. A search for asthma will thus also find an article indexed as *status asthmaticus* but the latter will be less weighted because indexing is said to be exploded.

^d^Some documents are indexed by a National Library of Medicine indexer; this is referred to as manual indexing. Other documents are indexed by text mining tools, which is referred to as automatic indexing. Manual indexing is considered to be more accurate and efficient than automatic indexing.

### Objectives

The goals of this study are to (1) determine which formula (A or B) is associated with the greatest topical relevance and (2) adjust the ranking formula’s criteria to meet the target end users’ needs more closely.

## Methods

### Overview

To evaluate the ranking formulae, we conducted formative user testing, which consisted of directly observing users using the tool in a controlled situation. It is a well-known method used in human factors to collect user behaviors and identify their needs [24].

Here, the user tests were conducted in two steps:

1. User evaluations of the two ranking formulae: the participants had to rate the relevance of search results produced by the two ranking formulae, while justifying their ratings. This step enabled us to determine which formula was associated with the greatest topical relevance.
2. Data collection for improving the ranking formulae: the participants had to rate the ranking criteria used in both formulae A and B and some additional criteria in terms of establishing an article’s relevance (from the most important criterion to the least important). Coupled with the users’ verbalizations when rating relevance, these data enabled us to adjust the ranking formula’s criteria and thus develop a formula that, in principle, would match professionals’ needs more closely.

### Step 1: Comparison of the 2 Ranking Formulae

#### Data Collection

LiSSa.fr [25] search logs were searched to identify the most frequent queries made by users. Among them, 2 were selected by a GP from the project consortium for their potential clinical value for the participants. Half of the participants (10/20, 50%) used the search query 1 (*treatment-resistant depression*) and the other half used search query 2 (*sleep apnea syndrome*; Table 2).

To compare the performances of ranking formulae A and B, each participant successively evaluated the search results generated by formulae A and B for the same query. The order of presentation of the formulae was counterbalanced to avoid order effects (Table 2). Participants were asked to perform their search query with LiSSa and then rate the relevance of the first 10 search results on a 5-point Likert scale ranging from 1 (not at all relevant) to 5 (highly relevant). For each result, the users had to justify their choice (eg, the article was too old or off-topic). The verbalizations for each rating were recorded.

After using both formulae, the users were asked to rate their overall level of satisfaction on a 7-point Likert scale ranging from 1 (“I am not at all satisfied with the search results”) to 7 (“I am fully satisfied with the search results”). For greater discriminative power, we chose to use a 7-point scale.

**Table 2:** Distribution of the participants according to the order in which the formulae and the predetermined search queries were presented (N=20).

Order of tested formula and predetermined search query		General physician participants, n (%)	Registrar participants, n (%)
**Tested formula A, then B**			
	Query 1	3 (15)	2 (10)
	Query 2	2 (10)	3 (15)
**Tested formula B, then A**			
	Query 1	2 (10)	3 (15)
	Query 2	3 (15)	2 (10)

#### Data Analysis

To check whether the *query* (query 1 vs query 2) and *type of participant* (GPs vs registrars) variables did not have an effect, a Mann–Whitney *U* test for independent samples was performed on the difference in scores between the formulae A and B.

To compare the user-perceived relevance for each formula, a Mann–Whitney *U* test for matched samples was performed on the three data sets:

- The scores given to the first 10 articles.
- The normalized discounted cumulative gain (NDCG) [34] was calculated from article scores. NDCG is an equation that calculates a score between 0 and 1; it evaluates the relevance of the article ranking using the scores given by the participants. Hence, the NDCG is close to 1 when the highest-rated articles are presented before the lowest-rated articles and, on the contrary, is close to 0 when the formula presents low-rated articles first and high-rated articles last.
- The overall satisfaction score awarded by the user at the end of the testing.

All statistical analyses were performed using R software (R Foundation for Statistical Computing) [35]. The threshold for statistical significance was set at *P*<.05 in all tests.

Participants’ verbalizations when justifying their scores were thematically analyzed [36]. This analysis enabled us to identify the strengths and weaknesses of each ranking formula, based on positive or negative comments. Each theme was counted once for each participant.

### Step 2: Prioritization of the Ranking Criteria

#### Data Collection

To improve the relevance of the ranking formulae and refine the criteria and their respective weightings, the participants were shown a list of criteria on separate cards, with the name of the criterion on one side and its explanation (that the user could consult, if required) on the other side (Table 3). The criteria were presented in random order for each participant. Additional explanations were provided upon request. The list contained both the criteria already included in formulae A and B and several other potentially relevant criteria.

The users were asked to classify the criteria by order of importance and to justify their choices. The justifications for each criterion were noted by specifying the item’s valence (positive or negative comments).

**Table 3:** List of the criteria shown to the participants.

Name	Explanation
Title	The keyword is present in the article’s title.
Subtitle	The keyword is present in the article’s subtitle.
Author keywords	The keyword is present in the author keywords.
Abstract	The keyword is present in the article’s abstract.
Major MeSH^a^ term	The keyword is present in the major MeSH term.
Minor MeSH term	The keyword is present in the minor MeSH term.
Exploded indexing or not^b^	Points are awarded if the indexing is not exploded (the keyword is the same as the MeSH term) vs exploded indexing (the keyword is found among the narrower MeSH terms).
Manual or automatic indexing	Points are awarded if the indexing is manual (performed by a National Library of Medicine indexer) rather than automatic (performed by text mining).
Association with a qualifier	Points are subtracted if the indexing qualifier is specified: for example, with *asthma/diagnosis*, the article will deal only with the diagnosis of asthma and not with asthma in general.
Year of publication	Points are awarded as a function of the article’s year of publication: the more recent it is, the more points it will be awarded.
Type of publication	Points are awarded if the article is a literature review, a good practice guideline, a consensus statement, a directive, or a meta-analysis.
Presence of an abstract	Points are awarded if an abstract in French is directly available on LiSSa (ie, without having to visit the journal’s website).
The journal’s importance	Points are awarded as a function of the journal’s impact.

^a^MeSH: Medical Subject Headings.

^b^The MeSH thesaurus contains qualifiers that can be linked to each keyword to make it more *precise*. For example, the index entry *asthma* can be specified by the qualifier *diagnosis* (*asthma/diagnosis*), to tell the reader that only the diagnosis of asthma is addressed in the article, and not its other aspects (treatment, complication, etc).

#### Data Analysis

We analyzed the classification of the ranking criteria by calculating the mean and median ranks for each criterion. Kendall *W* was used to evaluate the degree of interrater agreement.

Participants’ verbalizations were analyzed to characterize each criterion’s positive qualities (ie, why the user wanted to include it in the ranking formula) or negative qualities (ie, why it should not be taken into account or only partly in the formula).

### Test Participants

Calls for participation were made by the Department of General Practice and Family Medicine of the University of Lille by email to recruit GPs. Announcements were made during registrar classes, and calls for participation were posted in discussion groups and on social media pages to recruit hospital registrars. The only recruitment criterion was the profile of the participant (GP or registrar).

A total of 10 GPs and 10 registrars (ie, LiSSa’s target users) participated in the tests. They volunteered to participate, and no compensation was paid for their participation.

All sessions were filmed and subsequently analyzed offline by a usability engineer. The participants accessed LiSSa via a computer with an internet connection.

### Ethics Consideration

This study is a human and social science study. The French law governing ‘research involving the human person’ exempts human and social science studies from requiring approval from an ethics committee. Written informed consents were obtained from each participant before they took part in the study.

## Results

### Participant Characteristics

The characteristics of the participants are shown in Table 4.

**Table 4:** Participant characteristics.

Participant number	Profile	Age (years)	Number of years of practice (including internship semesters for registrar)	Self-reported frequency of use of a search engine
P1	GP^a^	29	2	Frequently
P2	GP	28	0	Frequently
P3	GP	30	2.5	Frequently
P4	GP	55	26	Frequently
P5	GP	56	29	Frequently
P6	GP	68	30	Frequently
P7	GP	53	16	Frequently
P8	GP	53	25	Not often
P9	GP	55	27	Frequently
P10	GP	33	5	Frequently
P11	Registrar	24	0.5	Never
P12	Registrar	26	0.5	Never
P13	Registrar	28	4	Never
P14	Registrar	26	1.5	Frequently
P15	Registrar	30	4	Not often
P16	Registrar	26	2	Frequently
P17	Registrar	28	1.5	Frequently
P18	Registrar	25	1.5	Not often
P19	Registrar	31	4.5	Frequently
P20	Registrar	29	5	Not often

^a^GP: general practitioner.

### Step 1: Comparison of the 2 Ranking Formulae

Our statistical analysis did not show a significant effect of *query* (treatment-resistant depression vs sleep apnea syndrome; *W*=4935; *P*=.87). Similarly, *the type of participant* (GPs vs registrars) did not have a significant effect (*W*=5071.5; *P*=.86). Therefore, a single user group (all 20 participants) was considered in the subsequent statistical tests.

Statistical tests showed that formula B was preferred to formula A with regard to all 3 end points (Table 5).

**Figure 2:**
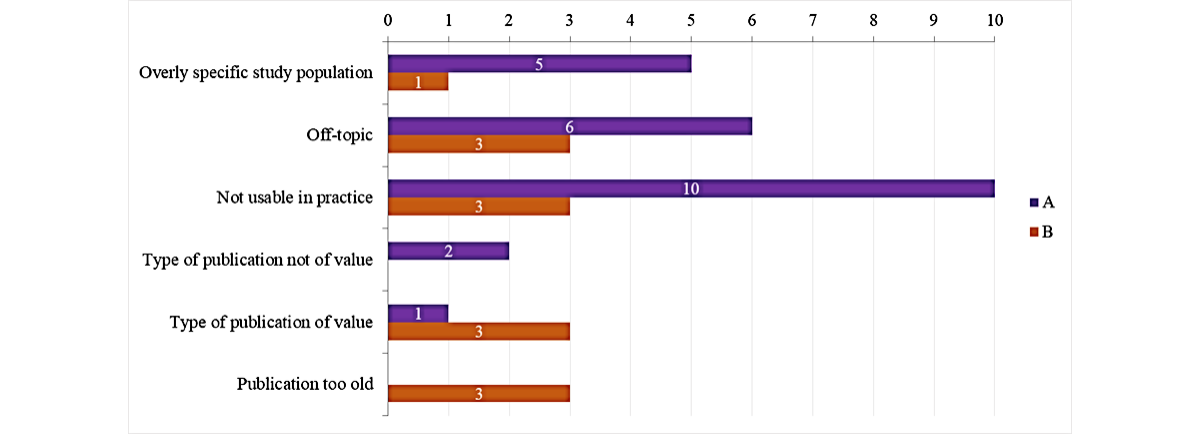
Types of negative verbalization about the articles for formula A or formula B; the number of participants is stated.

The analysis of the participants’ verbalizations confirmed this finding (Figure 2). Formula A attracted more negative comments: more participants expressed that the articles were not useful in practice, off-target, or too specific to a given population. Concerns about an article’s recentness were rarely expressed, although 15% (3/20) of the participants thought that at least one article presented by formula B was too old (Figure 2).

**Figure 3:**
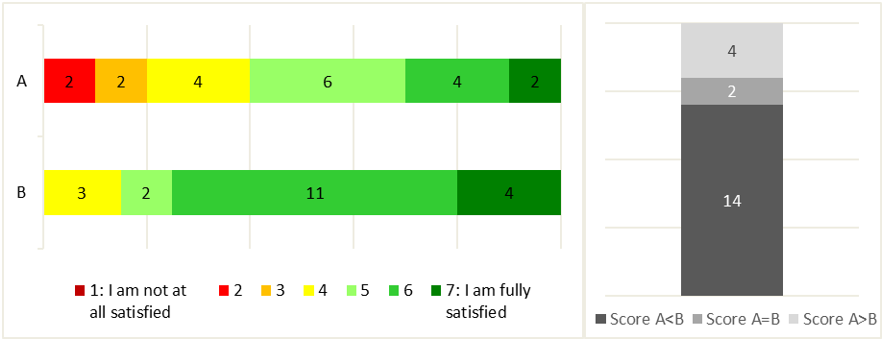
Distribution of the overall satisfaction score for formulae A and B (left panel), and the number of participants who gave formula A a higher, equal, or lower score than formula B (right panel).

Most participants (14/20, 70%) preferred formula B, notably because the articles’ topics were general and did not focus on a specific population (Figure 3). However, 30% (3/10) of GPs and 10% (1/10) of registrars preferred formula A because formula B presented trivial articles that taught them nothing new.

This phase of the evaluation prompted us to conclude that formula B best met participants’ expectations.

**Table 5:** The mean and median ranking scores, the normalized discounted cumulative gain (NDCG), and overall satisfaction scores for formulae A and B (N=20 participants).

	Formula A	Formula B	*W* value	*P* value
Main ranking score, median (IQR), out of 5	3.57 (4-2.5)	3.82 (4-3.5)	3518.5	.02
Main NDCG, median (IQR), out of 1	0.87 (0.95-0.83)	0.97 (0.99-0.94)	7	.01
Overall satisfaction score, median (IQR), out of 7	4.7 (5-4.6)	5.8 (6-5.6)	27.5	.01

### Step 2: Prioritization of the Ranking Criteria

In step 1, dealing with the formulae and search result scores enabled users to evaluate the formulae’s strengths and weaknesses. Because of the criteria ranking process, step 2 refined the users’ needs by moving out of the context of the present formulae and predetermined search queries. This part of the study enabled us to adjust formula B and thus make it more closely match the users’ needs. Of the 20 participants, 2 (10%) did not perform this step; hence, 18 (90%) participants (9 GPs and 9 registrars) prioritized the criteria.

The participants’ mean and median criterion rankings are shown in Table 6.

**Figure 4:**
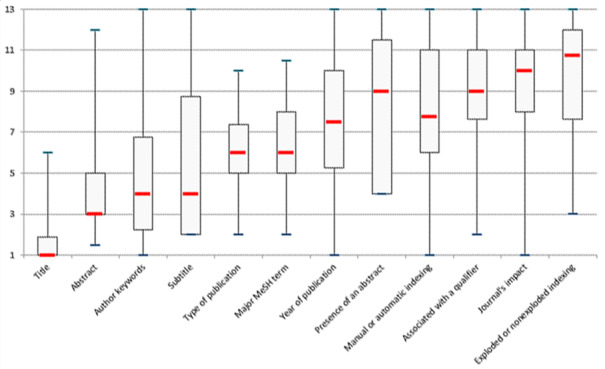
Boxplots of the scores (from 1 to 13) for each criterion. MeSH: Medical Subject Headings.

Given that the absence of an effect of *participants* had not been demonstrated for this data set, GPs and registrars were considered separately. There was a low but statistically significant degree of agreement among the GPs (*W*=0.46; *P*<.001; *r*=.39) and registrars (*W*=0.35; *P*<.001; *r*=.27). Despite this low agreement, clear trends emerged in the criteria rankings (Figure 4):

- Participants considered the *title* to be the most representative relevance criterion; it was one of the elements they looked at first, and it was thought to reflect the research rather well.
- After the title, the users looked for the presence of the query keywords in the *Abstract, author keywords*, and *subtitle*.
- Publication type was an important criterion because it enabled the selection of the most reliable articles (ie, those with a higher level of evidence). Participants gave low ratings to the editorials and letters.
- The *year of publication* is controversial. Some users judged it to be important because it reflected the latest advances, whereas others considered it to be highly dependent on the topic of the search query. Older articles are still used as benchmarks for practice in some fields.
- The remaining criteria were judged to be of secondary importance, albeit occasionally of value in differentiating between 2 articles with the same score. For example, articles describing more general studies were preferred to those describing more specific studies (*exploded indexing* and *associated with a qualifier*).

**Table 6:** Mean and median criterion ranks (n=18 participants).

Criterion	Mean rank	Median rank (IQR)
Title	1.8	1 (1.87-1)
Abstract	4.5	3 (5-3)
Author keywords	5.0	4 (6.75-4)
Subtitle	5.7	4 (6.75-2.25)
Type of publication	6.1	6 (7.38-5)
Major MeSH^a^ term	6.5	6 (8-5)
Year of publication	7.8	7.5 (10-5.25)
Presence of an abstract	8.1	7.8 (11.5-4)
Manual or automatic indexing	8.1	9 (11-6)
Associated with a qualifier	8.6	9 (11-7.62)
The journal’s impact	9.3	10 (11-8)
Exploded or nonexploded indexing	9.9	10 (12-7.63)
Minor MeSH term	9.9	10.8 (11.75-9)

^a^MeSH: Medical Subject Headings.

## Discussion

### Principal Findings

The overall objective of this paper is to present the value of using the user testing method to collect both quantitative and qualitative data, and to actively involve end users through an example of the evaluation of sorting formulae. A total of 2 formulae for ranking search results were evaluated to design a ranking formula that met the needs and expectations of the GPs and hospital registrars.

The first part of the study (scoring the relevance of both formulae’s search results) enabled us to compare the respective ranking efficiencies and participant preferences. Of the 20 users, 14 (70%) preferred formula B. These users liked articles that formed a consensus in the field, that is, reviews and meta-analyses that; for example, contained peer-approved definitions. Case reports and publications on highly specific elements (eg, a subcategory of patients) were judged to be of little use in practice. Of the 20 participants, 4 (20%; 3 GPs and 1 registrar) preferred formula A; the search results were more recent (ie, covering the latest theories or discoveries), although the level of evidence was lower. It is noteworthy that the more experienced GPs tended to be less interested in general articles because the latter taught them nothing new about their practice. A single ranking formula cannot meet all possible needs and expectations, so the user should be given a means to personalize the interface [37].

The second part of our study generated data on the perceived importance of the ranking criteria. Unquestionably, the ranking criteria judged by the participants to be the most valuable were those related to text data, that is, the match between the article’s metadata and the user’s search query. Thus, criteria such as *title*, *abstract*, *author keywords*, and *major MeSH terms* were often rated as the most important. The other criteria (ie, *the presence of an abstract*, *affiliation with a qualifier*, *and exploded indexing*) were judged to be useful, albeit mainly for differentiating between articles that already met the other criteria. The *type of publication* criterion was considered interesting because it highlighted the most reliable articles. Finally, the *year of publication* was a controversial criterion, the usefulness of which depended on the user’s search purpose.

In view of the results, formula B was selected for further study. Owing to our analysis of the verbalizations given during the formulae and criteria ranking steps, we were able to produce several adaptations of this ranking formula to better meet the users’ needs (Table 7). Each proposal was discussed between the Clinical Investigation Centre for Innovative Technology evaluation team and the design team of D2IM until they eventually reached a consensus. The opportunities for improvement, justifications, and state of implementation are listed in Table 7.

The main changes were the addition of the *keyword in the abstract* criterion and modification of the *type of publication* criterion. In addition to promoting consensus articles in a given field (eg, meta-analyses and literature reviews), the *type of publication* criterion downgrades articles that have little informative or scientific value for the reader (errata, questions and answers, personal narratives, etc). Another improvement discussed with the project partners was to provide the option to personalize LiSSa’s relevance ranking formula; for example, by manually adding customized search criteria or by automatically learning from users’ data to determine their preferences. Another approach for creating ranking formulae is machine learning based on the analysis of large quantities of user data [14]. Ultimately, it would be interesting to assemble this type of data for LiSSa and thus determine whether machine learning–based ranking formulae would differ from the formulae assessed in this study.

**Table 7:** Adaptations of the ranking formula, the associated justifications, and their state of implementation.

Criterion	Opportunity for improvement	Justification	State of implementation
Abstract	Take account of the keyword’s presence in the abstract.	Currently, the abstract is not considered at all, even though (on average), it was the second most important criterion, right after the title. However, the abstract is less strictly controlled than the author keywords and the MeSH^a^ indexing, giving it less weight that the latter.	In total, 3 points have been attributed to this criterion.
Subtitle	Lower is the weight attributed to the keyword’s presence in the subtitle, relative to its presence in the title.	The subtitle had the same weighting as the title (ie, 10) but was judged to be less important by the participants because it was less useful.	The number of points attributed to this criterion has dropped from 10 to 8.
Type of publication	Add a subcriterion to downgrade types of publication judged to be irrelevant by the users.	The *type of publication* criterion in formula B favors certain types of publication. The users recommended downgrading the types of publication of little practical interest for the users (eg, editorials, errata, historical articles, and letters).	A subcriterion had been added to the *type of publication* criterion. As well as awarding 3 points to certain publications, it removed 1 point for errata, questions and answers, personal accounts, portraits, commentaries, historical articles, editorials, letters, and case reports.
Associated with a qualifier	Promote subject headings without a qualifier, except when the keyword is a qualifier.	To prioritize articles that generally address the search subject in first search results, adding the *associated with a qualifier* criterion was recommended. Thus, subject headings without a qualifier will be favored, except when the qualifier is also one of the user’s keywords.	One point is added when the subject heading is not associated with a qualifier.
The journal’s impact	Add this criterion but do not give it much weight.	This criterion is not of major importance to users but can be useful for differentiating between 2 articles with the same score. It was recommended that this criterion should be taken into account when calculating the scores but should not be given much weight.	This item has not yet been incorporated into the LiSSa database. At present, this information is available for only 30% of articles; it will therefore be necessary to determine the relevance of integrating this criterion into the formula.
Operation of the ranking formula	Add the points awarded for the *title* and m*ajor or minor MeSH term* criteria.	During the tests, some publications considered by the participants to be off-topic were listed in the top search results (eg, an article on bipolar depression for the query on *treatment-resistant depression*). To limit the risk of seeing off-topic publications in the top search results, it was recommended to add points awarded for the *title* and *major or minor MeSH term* criteria.	This recommendation needs to be tested because it might have a negative effect on ranking the search results; it might overprioritize the articles with a large number of indexed keywords (>20, in some cases), relative to articles with few keywords.

^a^MeSH: Medical Subject Headings.

### Strengths and Limitations

The main advantage of formative assessment through user testing is that the study data are useful in the design process. The first step (where participants were asked to score the search results) enabled them to become familiar with the types of articles suggested by LiSSa and to think of the criteria that were important to them when judging an article’s relevance. As the rating of an article had to be justified, users had to become conscious of their judgment criteria. This first step also enabled us to identify the strengths and weaknesses of existing formulae. In step 2, the criteria ranking and the participants’ justifications helped us determine which criteria most strongly influenced the target users’ perception of relevance. When coupled with the strengths and weaknesses detected in step 1, these data enabled us to adjust the ranking formula’s criteria, and thus to develop a formula that should better meet health care professionals’ needs. This combined methodology allowed us to evaluate the formulae’s performance, collect user needs and habits, and evaluate the relevance of the articles found by the search engine.

In a user-centered design process, iterative evaluation during the design phase helps improve the tool before the final evaluation [18]. In contrast to more common methods [4,8], user testing is a relevant way to look for user inputs in the design of the article ranking formula. Typical methods for evaluating search engine relevance generally compare several ranking formulae; for example, a new ranking method against an old one [14,15]. In these 2 articles, the method used involved a large number of judges, which ensured good robustness of the results. The aim of comparing 2 ranking methods was achieved, but no additional information was obtained to improve the relevance of the results. These methods did not capture the reasons why the search results were judged to be more relevant by the participants or the criteria that participants used to assess relevance. Collecting and analyzing participants’ verbalizations during user testing allowed us to understand the strengths and weaknesses of the tested formulae and to look for improvements suggested directly by end users. Even if this method is applied here in the context of a French language health scientific literature search engine, it can be used for any type of ranking formula.

Nevertheless, this study had some limitations. Formative assessments generate data to improve a formula’s design but do not validate a formula per se. Several criteria must be fulfilled for reliable and robust validation: the size of the test collection, the number of judges, the number of queries, and so on, [7,38] which a formative evaluation cannot fulfill. In total, 10 GPs and 10 registrars participated in this study. Moreover, as LiSSa is already on the web, user feedback shows that other health care professionals are using the tool (nurses, specialist physicians, physiotherapists, etc): different users might have different needs. The results are not generalizable and do not validate the formula. Therefore, a larger-scale evaluation with a larger number of participants and a broader range of user profiles is needed to evaluate and validate the final version of the formula.

Finally, a significant limitation of all approaches aimed at improving search result ranking formulae relates to the quality and availability of metadata. The level of topical relevance does not depend solely on identification and weighting; for each criterion, the data must be tagged for each article in the database. If this is not performed, the addition of a criterion may have very little impact on the result ranking or may even degrade the quality of the result ranking. During this evaluation, the criteria used were based on metadata available in the LiSSa database. We can assume that a change in the available metadata would have opened new opportunities for ranking search results, and therefore, would have impacted the study results. During our tests, we asked the participants whether criteria other than those presented should be added. Several criteria had been suggested (ie, *type of population*, *medical specialty*, *methodology*, etc), but none can yet be considered for inclusion because the related metadata are not available or are not of high enough quality or coverage for reliable incorporation in the formula. This was also the case for the criteria presented during the study. Some criteria presented during the second step of the study were interesting to users but could not be implemented directly after the evaluation. For example, *journal importance* metadata were available for >30% of the articles included in the LiSSa database, which prevented us from implementing this criterion. Nevertheless, the identification of new criteria has challenged the design team to add metadata and complete the formula using new criteria in the near future.

The LiSSa database contains various types of publications from thousands of journals and hundreds of publications. Each publisher has its own rules for tagging articles because they do not address the same indexing objectives [39]. Therefore, the creation of a database of over a million articles is already a challenge, particularly with regard to harmonizing metadata of different types and formats. Thus, the reuse of metadata is an objective, within which the ranking of results is just one of many challenges. This reveals the need for a true debate among all stakeholders (ie, publishers, institutions, and users) about standardized indexing that meets various objectives (eg, ranking and archiving).

### Conclusions

To conclude, LiSSa is a tool intended for practitioners who are not specialized in scientific research and who do not speak English. This study highlights the need of these end users to improve the topical relevance of the first top-ranked results. The assessment of the LiSSa search engine’s result ranking formulae enabled us to draw a list of recommendations for a ranking formula that would meet the ranking needs of GPs and hospital registrars. In the next step of the project, we will assess the relevance and appropriateness of the redesigned ranking formula with regard to user needs and expectations. To this end, we shall conduct tests with a new panel of users that includes more types of health care professionals.

## Abbreviations

D2IM: Department of Medical Informatics and Information
GP: general practitioner
MeSH: Medical Subject Headings
NDCG: normalized discounted cumulative gain
NLM: National Library of Medicine
